# Long term persistence of coronavirus SARS-CoV-2 infection

**DOI:** 10.31744/einstein_journal/2021RC6369

**Published:** 2021-11-24

**Authors:** João Renato Rebello Pinho, Ketti Gleyzer de Oliveira, Roberta Sitnik, Maira Marranghello Maluf, Pedro Henrique Sebe Rodrigues, Rúbia Anita Ferraz Santana, Eliane Rosseto Welter, Ophir Irony

**Affiliations:** 1 Hospital Israelita Albert Einstein São Paulo SP Brazil Hospital Israelita Albert Einstein, São Paulo, SP, Brazil

**Keywords:** COVID-19, SARS-CoV-2, Infecções Coronavírus, Coronavírus

## Abstract

During the COVID-19 pandemic, a case of a long-term persistence of SARS-CoV-2 infection (from March 26 to May 20, 2020) was identified at a private hospital in São Paulo, SP, Brazil. The long-term positivity for SARS-CoV-2 in reverse transcriptase polymerase chain reaction tests of a patient diagnosed with COVID-19 suggests, at least part of patients who recovered, may still carry and transmit the SARS-CoV-2 virus. This fact emphasizes the importance of having at least two negative reverse transcriptase polymerase chain reaction test results for SARS-CoV-2. Serological assays were not particularly helpful in the case described, since the patient had very low antibodies titers at the end of the follow-up period. Low viral loads may not be detected by current molecular methods, leading to wrong conclusions regarding viral clearance.

## INTRODUCTION

Coronaviruses are important human and animal pathogens. At the end of 2019, a novel coronavirus was identified as the cause of a cluster of pneumonia cases in the city of Wuhan, in the province of Hubei, China.^(1)^ These were the first cases of the disease now known as coronavirus disease 2019 (COVID-19), and its etiological agent was named severe acute respiratory syndrome coronavirus 2 (SARS-CoV-2). The fast spread of COVID-19 resulted in an epidemic throughout China, which was followed by an increasing number of cases around the world.^(2)^ Therefore, on January 30, 2020, the World Health Organization (WHO) issued a level 3 global SARS-CoV-2 infection alert.^(3,4%)^

Clinical manifestations of COVID-19 can vary widely, ranging from mild or asymptomatic, especially in young adults and children, to severe presentation, including pneumonia, septic shock and respiratory failure.^(4,5%)^ Pneumonia seems to be the most frequent severe manifestation of infection and is characterized by fever, cough, dyspnea and bilateral lung infiltrates on chest imaging tests. However, other manifestations, such as upper respiratory tract symptoms, myalgia, diarrhea, and smell or taste disorders are also common. COVID-19 is often severe among elderly patients and patients with medical comorbidities.^(6–8%)^

During COVID-19 pandemic, a case with long-term persistence of SARS-CoV-2 (March 26 to May 20, 2020) was identified at a private hospital located in São Paulo, SP, Brazil. This study was approved by local ethics committee (CAAE: 34866420.3.0000.0071, opinion number 4.159.562). The patient involved signed an Informed Consent form.

## CASE REPORT

A 23-year-old woman presented at the hospital on March 24, 2020, with a history of exposure to a close contact (her partner) with positive SARS-CoV-2 reverse transcriptase polymerase chain reaction (RT-PCR) test results. The patient reported fever lasting 4 days, odynophagia, nasal congestion, mild dry cough and fatigue, but no dyspnea. She was on bupropion hydrochloride and naltrexone for weight control (normal body mass index), had a history of pancreatitis of unknown etiology (in 2015) and no history of significant infections. Her immunization schedule was up to date, with the exception of influenza H1N1 vaccine.

Upon admission, her vital signs revealed fever with axillary temperature of 38°C, systolic blood pressure (SBP) of 89mmHg, diastolic blood pressure (DBP) of 61mmHg, mean arterial pressure of 70mmHg, pulse rate of 114bpm (*i.e.,* high), respiratory rate (RR) of 18rpm and oxygen saturation (SatO_2_) of 96% at room air. Some laboratory tests were requested (Table 1).

**Table 1:** Automated complete blood count and other tests during infection

Admission Days after onset of symptoms	March 24	March 29	April 10	April 26	May 3	May 4	May 11	May 20	Reference ranges
	Day 5	Day 10	Day 22	Day 38	Day 45	Day 46	Day 53	Day 62	
Erythrocytes, 10^6^/uL	3.8*	3.6*	4.05	3.25*	3.38*	3.41*	3.94	3.7*	3.90-5.00
Hemoglobin, g/dL	12.4	11.7*	13.0	10.7*	10.7*	11.2*	12.8	11.8*	12.0-15.5
Hematocrit,%	37.0	34.8*	37.8	31.6*	32.6*	33.7*	36.9	34.3*	35.0-45.0
MCV, fL	97.4	96.7	93.3	97.2	96.4	98.8*	93.7	92.7	82.0-98.0
MCH, pg	32.6	32.5	32.1	32.9	31.7	32.8	32.5	31.9	26.0-34.0
MCHC, g/dL	33.5	33.6	34.4	33.9	32.8	33.2	34.7	34.4	31.0-36.0
RDW, %	13.8	13.0	12.2	12.6	13.0	12.4	11.6	11.5	11.5-16.5
Leucocytes,mL	6,290	3,330*	4,860	6,950	5,520	4,760	9,780	6,410	3,500-10,500
Neutrophils,mL	4,378	1,812	2,308	4,198	2,666	2,723	7,032	3,923	1,700-8,000
Eosinophils,mL	182	30*	102	132	237	119	108	128	50-500
Basophils,mL	19	10	29	49	28	38	39	19	0-100
Lymphocytes,mL	1,088	1,129	2,070	2,029	2,131	1,461	2,083	1,731	900-2,900
Monocytes,mL	623	350	350	542	458	419	518	609	300-900
Platelets,mL	395,000	319,000	410,000	258,000	336,000	393,000	363,000	313,000	150,000-450,000
Mean platelet volume, fL	8.5	8.3	9.7	9.3	9.0	8.9	9.5	9.0	6.5-15.0
C-reactive protein, mg/L	2.3	6.2*	<0.3	18.7*	1.4	0.9	0.4	2.2	≤5.0

* Results outside the reference range.

MCV: mean corpuscular volume; MCH: mean corpuscular hemoglobin; MCHC: mean corpuscular hemoglobin concentration; RDW: red blood cell distribution width.

**Figure 1:**
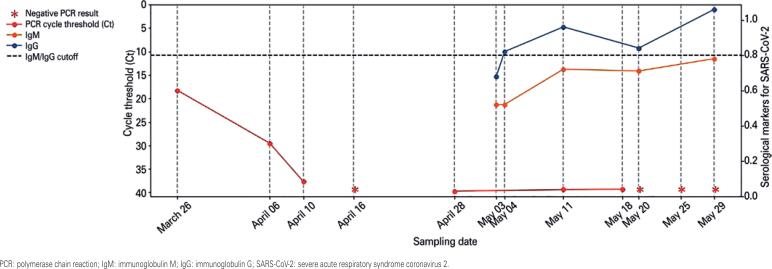
Consecutive results of tests conducted with nasopharyngeal samples. SARS-CoV-2 reverse transcriptase polymerase chain reaction cycle threshold values are shown in red. Serological markers for COVID-19 are shown in blue (immunoglobulin M) and orange (immunoglobulin G)

Among the presumptive diagnoses, the main was non-specific acute upper airway infection unrelated to chronic disease. For epidemiological purposes, nasopharyngeal and oropharyngeal specimens were collected for SARS-CoV-2 RT-PCR testing. The requesting physician recommended home isolation for 14 days, and oriented the patient to return if symptoms got worse. Positive SARS-CoV-2 RT-PCR test results were obtained on March 26, 2020, with a cycle threshold (Ct) value of 18.29. Results obtained in different SARS-CoV-2 RT-PCR tests are shown in figure 1. RT-PCR tests were run using Cepheid and Mobius assays.

On March 29, 2020, the patient returned to the hospital complaining of persistent fever for 6 days and dyspnea for the past 2 days. She had taken dipyrone and paracetamol. On this occasion, physical examination revealed the following vital signs: axillary temperature of 38°C, SBP of 98mmHg, DBP of 63mmHg, pulse rate of 100bpm, SatO_2_ of 98% and RR of 18rpm.

**Figure 2:**
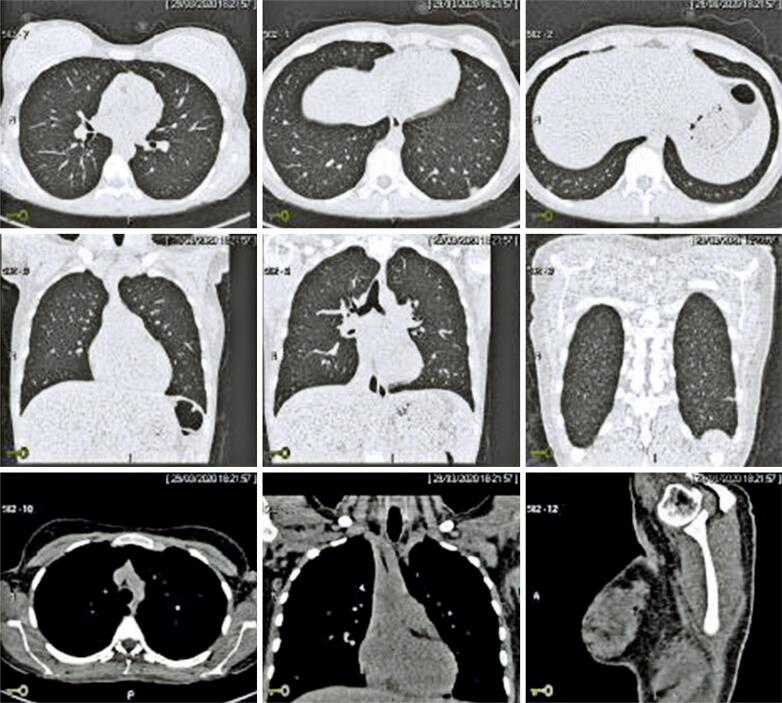
Multislice helical high-resolution computed tomography with no use of intravenous contrast. Chest and lungs images suggestive of pulmonary infection

High resolution computed tomography (CT) of the chest and lungs and laboratory tests were performed. Laboratory tests revealed mild leukopenia with no signs of severity. Computed tomography revealed rare ground-glass opacities in the lungs, particularly in the lung periphery and lower lobes, and mild opacity in the posterolateral aspect of the posterior segment of the lower left pulmonary lobe. Nonspecific findings suggestive of incipient inflammation/infection were also detected. The estimated extent of pulmonary involvement based on CT images was less than 50% (Figure 2). Nasopharyngeal and oropharyngeal secretion specimens were collected for SARS-CoV-2 RT-PCR testing to monitor the infection. The patient was oriented to remain in home isolation for another 14 days.

The patient reported significant clinical improvement during home isolation, presenting mild anosmia. However, SARS-CoV-2 RT-PCR tests results remained positive for 16 days (Ct values of 29.53 and Ct of 37.69 on April 6 and April 10, 2020, respectively).

During the patient follow-up, the first SARS-CoV-2 RT-PCR test with undetected result was on April 16, 2020.

Ten days later (April 26, 2020), the patient returned to the hospital with significant recurrence of symptoms. She reported fever in the past 6 days, myalgia and abdominal pain, but no respiratory symptoms. She also reported anosmia and macular lesions on skin. Physical examination and abdominal ultrasound revealed no abnormalities. Other laboratory tests (SARS-CoV-2 RT-PCR, SARS-CoV-2 serology and complete blood count) were ordered and the patient was discharged home. On April 28, 2020, RT-PCR test results were again positive, with a Ct value of 39.72.

The patient returned to the hospital on May 3, 2020 reporting persistent fever in the past 13 days, cough, progressive respiratory distress, back pain for one day and intermittent shortness of breath. Physical examination revealed no abnormalities. Chest CT was repeated and no focal pulmonary opacities suggestive of active infection were found.

RT-PCR and serological tests were repeated to investigate the progression of SARS-CoV-2 infection in this patient. Results of SARS-CoV-2 RT-PCR and serological tests performed during the follow-up period are shown in figure 1.

Other infectious etiologies, such as acute arbovirus, coxsackie A and B virus, cytomegalovirus, toxoplasmosis, Epstein-Barr virus and HIV infection, were investigated and ruled out. Screenings for autoimmune, endocrine, and metabolic diseases were also negative.

Little emphasis has been given to the follow-up of patients with oscillating positive tests results for SARS-CoV-2 RNA. As showed in the figure, the decrease in RT-PCR Ct values in this patient was very similar to patterns seen in many patients during the first two weeks of follow-up. The first negative PCR result was obtained within three weeks of the onset of symptoms. However, samples collected during the following month were positive. Consecutive negative results could only be obtained 56 days after the first positive result. Low viral load suggests the virus is not able to infect other patients. However, detection of viral RNA in three consecutive nasopharyngeal and oropharyngeal samples indicated at least some remnant RNA were present in this case.

## DISCUSSION

Lan et al., described positive RT-PCR test results 5 to 13 days after discharge, in four patients with COVID-19 who had met hospital discharge or quarantine discontinuation criteria in China (absence of clinical symptoms and radiological abnormalities and two negative RT-PCR test results).(9) These findings were corroborated by Wu et al., in a study reporting positive SARS-CoV-2 RT-PCR tests results in 10 out of 60 patients diagnosed with and treated for COVID-19 within 4 to 24 days after hospital discharge.^(10)^

## CONCLUSION

The long-term positivity for SARS-CoV-2 in reverse transcriptase polymerase chain reaction tests in the patient reported, who was sent home with diagnosis of COVID-19, suggests that at least part of patients who recover may still carry and possibly transmit the SARS-CoV-2 virus. Therefore, the importance of obtaining a minimum of two negative SARS-CoV-2 reverse transcriptase polymerase chain reaction test results must be emphasized. Serological assays were not particularly helpful in the case reported, since the patient had very low antibodies titers at the end of the follow-up period. Low viral loads may not be detected by current molecular methods, leading to wrong conclusions regarding viral clearance.

Given the catastrophic collapse of hospitals, further cohort studies with clinical and laboratory characterization of COVID-19 patients, and including consecutive SARS-CoV-2 RNA test results are warranted for deeper understanding and enhanced response to the COVID-19 pandemic. Improved patient characterization may translate into more effective management and epidemiological surveillance of COVID-19 cases outside the health system.

## References

[1] She J, Jiang J, Ye L, Hu L, Bai C, Song Y. 2019 novel coronavirus of pneumonia in Wuhan, China: emerging attack and management strategies. Clin Transl Med. 2020;9(1):19. Review. 10.1186/s40169-020-00271-z PMC7033263 32078069

[2] World Health Organization (WHO). WHO Director-General’s remarks at the media briefing on 2019-nCoV on 11 February 2020. Geneva: WHO; 2020 [cited 2020 Feb 12]. Available from: https://www.who.int/dg/speeches/detail/who-director-general-s-remarks-at-the-media-briefing-on-2019-ncov-on-11-february-2020

[3] World Health Organization (WHO). Novel Coronavirus (2019-nCoV): situation report - 10. Geneva: WHO; 2020 [cited 2020 Feb 2]. Available from: https://www.who.int/docs/default-source/coronaviruse/situation-reports/20200130-sitrep-10-ncov.pdf?sfvrsn=d0b2e480_2

[4] Sifuentes-Rodríguez E, Palacios-Reyes D. COVID-19: the outbreak caused by a new coronavirus. Bol Med Hosp Infant Mex. 2020;77(2):47-53. Review. 10.24875/BMHIM.20000039 32226003

[5] Shaker MS, Oppenheimer J, Grayson M, Stukus D, Hartog N, Hsieh EW, et al. COVID-19: pandemic contingency planning for the allergy and immunology clinic. J Allergy Clin Immunol Pract. 2020;8(5):1477-88.e5. Review. 10.1016/j.jaip.2020.03.012 PMC7195089 32224232

[6] Guan WJ, Ni ZY, Hu Y, Liang WH, Ou CQ, He JX, Liu L, Shan H, Lei CL, Hui DS, Du B, Li LJ, Zeng G, Yuen KY, Chen RC, Tang CL, Wang T, Chen PY, Xiang J, Li SY, Wang JL, Liang ZJ, Peng YX, Wei L, Liu Y, Hu YH, Peng P, Wang JM, Liu JY, Chen Z, Li G, Zheng ZJ, Qiu SQ, Luo J, Ye CJ, Zhu SY, Zhong NS; China Medical Treatment Expert Group for Covid-19. Clinical characteristics of coronavirus disease 2019 in China. N Engl J Med. 2020;382(18):1708-20. 10.1056/NEJMoa2002032 PMC7092819 32109013

[7] Zhou F, Yu T, Du R, Fan G, Liu Y, Liu Z, et al. Clinical course and risk factors for mortality of adult inpatients with COVID-19 in Wuhan, China: a retrospective cohort study. Lancet. 2020;395(10229):1054-62. Erratum in: Lancet. 2020;395(10229):1038. 10.1016/S0140-6736(20)30566-3 PMC7270627 32171076

[8] Liu Y, Yan LM, Wan L, Xiang TX, Le A, Liu JM, et al. Viral dynamics in mild and severe cases of COVID-19. Lancet Infect Dis. 2020;20(6):656-7. 10.1016/S1473-3099(20)30232-2 PMC7158902 32199493

[9] Lan L, Xu D, Ye G, Xia C, Wang S, Li Y, et al. Positive RT-PCR test results in patients recovered from COVID-19. JAMA. 2020;323(15):1502-3. 10.1001/jama.2020.2783 PMC7047852 32105304

[10] Wu J, Liu X, Liu J, Liao H, Long S, Zhou N, et al. Coronavirus disease 2019 test results after clinical recovery and hospital discharge among patients in China. JAMA Netw Open. 2020;3(5):e209759. 10.1001/jamanetworkopen.2020.9759 PMC7244988 32442288

