# Supplementary material for: Long term persistence of coronavirus SARS-CoV-2 infection
Source: Einstein (Sao Paulo). 2021 Nov 24;19:eRC6369. doi: 10.31744/einstein_journal/2021RC6369 (PMC8664288; doi:10.31744/einstein_journal/2021RC6369)
Supplement: Translation

# Translation: infecção persistente de longo prazo pelo coronavírus SARS-CoV-2

**Authors:** João Renato Rebello Pinho, Ketti Gleyzer de Oliveira, Roberta Sitnik, Maira Marranghello Maluf, Pedro Henrique Sebe Rodrigues, Rúbia Anita Ferraz Santana, Eliane Rosseto Welter, Ophir Irony

**Affiliations:** 1 São Paulo SP Brazil Hospital Israelita Albert Einstein, São Paulo, SP, Brazil

**Keywords:** COVID-19o, SARS-CoV-2, Infecções por coronavírus, Coronavírus

## Abstract

Durante a pandemia da COVID-19, um caso de persistência de longo prazo de infecção por SARS-CoV-2, de 26 de março a 20 de maio de 2020, foi identificado em um hospital privado localizado em São Paulo, SP, Brasil. A positividade de longo prazo para SARS-CoV-2 nos exames de reação em cadeia da polimerase via transcriptase reversa de uma paciente diagnosticada com COVID-19 sugere que parte dos pacientes que se recuperaram podem ser portadores e transmitir o SARS-CoV-2. Esse fato enfatiza a importância da obtenção de pelo menos dois resultados negativos para SARS-CoV-2 no exame de reação em cadeia da polimerase via transcriptase reversa. Os ensaios sorológicos não foram de grande utilidade no caso descrito, uma vez que a paciente apresentava baixos títulos de anticorpos no final do período de acompanhamento. Baixas cargas virais podem não ser detectadas pelos métodos moleculares vigentes, levando a conclusões equivocadas a respeito da eliminação do vírus.

## INTRODUÇÃO

Os coronavírus são patógenos importantes em seres humanos e animais. No final de 2019, um novo coronavírus foi identificado como causa de um cluster de casos de pneumonia na cidade de Wuhan, localizada na província de Hubei, na China.^(1)^ Esses foram os primeiros casos da doença atualmente conhecida como doença do coronavírus 2019 (COVID-19), e seu agente etiológico é o coronavírus da síndrome respiratória aguda grave 2 (SARS-CoV-2). A rápida disseminação da COVID-19 resultou em uma epidemia que afetou toda a China, seguindo-se de um número crescente de casos no mundo todo.^(2)^ Por esse motivo, a Organização Mundial da Saúde (OMS) emitiu um alerta global de nível 3 para essa infecção em 30 de janeiro de 2020.^(3,4)^

As manifestações clínicas associadas à COVID-19 podem variar bastante, desde uma apresentação leve ou assintomática, principalmente em adultos jovens e crianças, até uma apresentação mais grave, que inclui pneumonia, choque séptico e insuficiência respiratória.^(4,5)^A pneumonia parece ser a manifestação grave mais frequente da infecção, caracterizada por febre, tosse, dispneia e infiltrados pulmonares bilaterais em exames de imagem do tórax. Entretanto, outras manifestações, como sintomas associados ao trato respiratório superior, mialgia, diarreia e distúrbios do olfato e paladar, também são comuns. A COVID-19 costuma ser grave em pacientes idosos e com comorbidades médicas.^(6–8)^

Durante a vigência da pandemia, um caso de persistência de longo prazo do SARS-CoV-2, de 26 de março a 20 de maio de 2020, foi identificado em um hospital particular localizado em São Paulo, SP, Brasil. Este estudo foi aprovado pelo comitê local de ética (CAAE: 34866420.3.0000.0071, número do parecer 4.159.562). A paciente envolvida assinou um Termo de Consentimento Informado.

## RELATO DO CASO

Uma mulher de 23 anos procurou o hospital em 24 de março de 2020 com histórico de exposição a contato próximo (seu companheiro) com resultado positivo no exame de reação em cadeia da polimerase via transcriptase reversa (RT-PCR) para SARS-CoV-2. A paciente relatou febre que durava 4 dias, odinofagia, congestão nasal, tosse seca leve e fadiga, embora sem dispneia. A paciente estava em uso de cloridrato de bupropiona e naltrexona para controle de peso (índice de massa corporal normal), relatou quadro de pancreatite de etiologia desconhecida (ocorrida em 2015) e não tinha histórico de infecções prévias importantes. A paciente estava com o cronograma de vacinações em dia, com exceção da vacina contra influenza H1N1.

À admissão, apresentou os seguintes sinais vitais: febre com temperatura axilar de 38°C, pressão arterial sistólica (PAS) de 89mmHg, pressão arterial diastólica (PAD) de 61mmHg, pressão arterial média de 70mmHg, pulso de 114bpm (ou seja, valor aumentado), frequência respiratória (FR) de 18rpm e saturação de oxigênio (SatO_2_) de 96% ao ar ambiente. Foram solicitados alguns exames laboratoriais (Tabela 1).

**Tabela 1:** Contagem automática de hemograma completo e outros testes durante a infecção

Admissão Dias após o início dos sintomas	24 de março	29 de março	10 de abril	26 de abril	3 de maio	4 de maio	11 de maio	20 de maio	Faixas de referência
	Dia 5	Dia 10	Dia 22	Dia 38	Dia 45	Dia 46	Dia 53	Dia 62	
Eritrócitos, 10^6^/uL	3,8*	3,6*	4,05	3,25*	3,38*	3,41*	3,94	3,7*	3,90-5,00
Hemoglobina, g/dL	12,4	11,7*	13,0	10,7*	10,7*	11,2*	12,8	11,8*	12,0-15,5
Hematócrito, %	37,0	34,8*	37,8	31,6*	32,6*	33,7*	36,9	34,3*	35,0-45,0
VCM, fL	97,4	96,7	93,3	97,2	96,4	98,8*	93,7	92,7	82,0-98,0
HCM, pg	32,6	32,5	32,1	32,9	31,7	32,8	32,5	31,9	26,0-34,0
CHCM, g/dL	33,5	33,6	34,4	33,9	32,8	33,2	34,7	34,4	31,0-36,0
RDW, %	13,8	13,0	12,2	12,6	13,0	12,4	11,6	11,5	11,5-16,5
Leucócitos,mL	6.290	3.330*	4.860	6.950	5.520	4.760	9.780	6.410	3.500-10.500
Neutrófilos,mL	4.378	1.812	2.308	4.198	2.666	2.723	7.032	3.923	1.700-8.000
Eosinófilos,mL	182	30*	102	132	237	119	108	128	50-500
Basófilos,mL	19	10	29	49	28	38	39	19	0-100
Linfócitos,mL	1.088	1.129	2.070	2.029	2.131	1.461	2.083	1.731	900-2.900
Monócitos,mL	623	350	350	542	458	419	518	609	300-900
Plaquetas,mL	395.000	319.000	410.000	258.000	336.000	393.000	363.000	313.000	150.000-450.000
Volume plaquetário médio, fL	8,5	8,3	9,7	9,3	9,0	8,9	9,5	9,0	6,5-15,0
Proteína C-reativa, mg/L	2,3	6,2*	<0,3	18,7*	1,4	0,9	0,4	2,2	≤5,0

* Resultados fora da faixa de referência.

VCM: volume corpuscular médio; HCM: hemoglobina corpuscular média; CHCM: concentração da hemoglobina corpuscular média; RDW: red blood cell distribution width.

Entre as hipóteses diagnósticas, a principal foi infecção aguda inespecífica das vias aéreas superiores sem relação com doença crônica. Para fins de investigação epidemiológica, foram coletadas amostras naso e orofaríngeas para exame de RT-PCR para SARS-CoV-2. O médico recomendou isolamento doméstico por 14 dias e retorno caso os sintomas viessem a piorar. O resultado da RT-PCR para SARS-CoV-2, divulgado em 26 de março de 2020, foi positivo, com valor limiar de ciclo (Ct) de 18,29. Os resultados dos diversos exames de RT-PCR para SARS-CoV-2 podem ser visualizados na figura 1. Os exames de RT-PCR em tempo real foram realizados com os ensaios da Cepheid e Mobius.

**Figure 1:** Resultados de testes consecutivos realizados com amostras nasofaríngeas. Valor limiar de ciclo de reação em cadeia da polimerase via transcriptase reversa para SARS-CoV-2 (vermelho). Marcadores sorológicos para COVID-19 em azul (imunoglobulina M) e laranja (imunoglobulina G

Em 29 de março de 2020, a paciente retornou ao hospital com queixa de febre persistente por 6 dias e dispneia nos últimos 2 dias. Tinha feito uso de dipirona e paracetamol. Nessa ocasião, o exame físico revelou os seguintes sinais vitais: temperatura axilar de 38°C, PAS de 98mmHg, PAD de 63mmHg, pulso de 100bpm, SatO_2_ de 98% e FR de 18rpm.

A paciente foi submetida à tomografia computadorizada (TC) de alta resolução do tórax e pulmões e a novos exames laboratoriais. Os exames mostraram leucopenia leve, sem sinais de gravidade. A TC revelou a presença de raras opacidades em padrão de vidro fosco nos pulmões, mais evidentes na periferia e nos lobos inferiores, e opacidade leve na região posterolateral do segmento posterior do lobo pulmonar inferior esquerdo. Achados inespecíficos sugestivos de processo inflamatório/infeccioso incipiente também foram encontrados. O grau estimado do envolvimento pulmonar, com base na TC, foi de menos de 50% (Figura 2). Novas amostras de secreção da naso e orofaringe foram coletadas para RT-PCR para SARS-CoV-2, a fim de monitorar a infecção. A paciente foi orientada a se manter em isolamento doméstico por mais 14 dias.

**Figure 2:** Tomografia computadorizada de alta resolução helicoidal multislice, sem uso de contraste intravenoso. Imagens torácicas e pulmonares sugestivas de processo infeccioso pulmonar

A paciente apresentou melhora clínica significativa durante o período de isolamento doméstico, relatando apenas persistência de anosmia discreta. Entretanto, os resultados da RT-PCR para SARS-CoV-2 permaneceram positivos por 16 dias, com valores de Ct de 29,53 e 37,69 (dias 6 e 10 de abril de 2020, respectivamente).

Durante o seguimento da paciente, o primeiro resultado negativo na RT-PCR para SARS-CoV-2 foi em 16 de abril de 2020.

Dez dias depois, em 26 de abril de 2020, a paciente retornou ao hospital, com recorrência importante dos sintomas, queixando-se de febre nos últimos 6 dias, mialgia e dor abdominal, porém sem sintomas respiratórios. Outros sintomas relatados foram anosmia e aparecimento de lesões maculares na pele. O exame físico e a ultrassonografia de abdômen não apresentaram alterações. Novos exames foram solicitados (RT-PCR para SARS-CoV-2, sorologia para SARS-CoV-2 e hemograma completo), e a paciente teve alta. Em 28 de abril de 2020, o resultado da RT-PCR foi positivo, com Ct de 39,72.

A paciente retornou ao hospital no dia 3 de maio de 2020 com queixa de febre persistente nos últimos 13 dias, tosse, dificuldade respiratória progressiva, dor nas costas com 1 dia de duração e falta de ar intermitente. O exame físico não revelou nada digno de nota. A TC do tórax foi repetida e mostrou ausência de opacidades pulmonares focais sugestivas de processo infeccioso ativo.

Depois disso, novos testes sorológicos e RT-PCR para SARS-CoV-2 foram solicitados a fim de se estudar a evolução da infecção. Esses resultados estão apresentados na figura 1.

Outras etiologias infecciosas foram investigadas e descartadas, como infecção aguda por arbovírus, vírus coxsackie A e B, citomegalovírus, toxoplasmose, vírus Epstein-Barr e HIV. O rastreamento de doenças autoimunes, endócrinas, e metabólicas também foi negativo.

O acompanhamento de pacientes com oscilação de resultados positivos para presença de RNA de SARS-CoV-2 tem sido objeto de pouca atenção. A paciente em questão apresentou queda dos valores de Ct na RT-PCR, semelhante à que ocorre em muitos pacientes durante as primeiras duas semanas de acompanhamento. O primeiro resultado negativo na PCR foi obtido três semanas após o aparecimento dos sintomas, porém outras amostras coletadas durante o mês seguinte foram positivas. Resultados negativos consecutivos só foram obtidos após o 56º dia depois do primeiro resultado positivo. A baixa carga viral sugere que o vírus não é capaz de infectar outros pacientes. Entretanto, a detecção de RNA viral em três amostras consecutivas de secreção da naso e orofaringe mostrou que pelo menos alguns resquícios de RNA permaneciam no organismo da paciente descrita.

## DISCUSSÃO

Lan et al., descreveram resultados positivos em testes de RT-PCR realizados 5 a 13 dias após a alta em quatro pacientes com COVID-19 que preencheram os critérios de alta hospitalar ou suspensão da quarentena na China (ausência de sintomas clínicos e alterações radiológicas e dois resultados negativos no teste da RT-PCR).^(9)^ Estudo realizado por Wu et al., mostrou que dez de 60 pacientes com diagnóstico prévio de COVID-19 testaram positivo para SARS-CoV-2 na RT-PCR entre 4 e 24 dias após a alta hospitalar, corroborando os achados do primeiro estudo.^(10)^

## CONCLUSÃO

A positividade de longo prazo para SARS-CoV-2 no exame de reação em cadeia da polimerase via transcriptase reversa na paciente descrita, diagnosticada com COVID-19 sem necessidade de internação, sugere que pelo menos parte dos pacientes que se recuperam podem ser portadora e transmitir o SARS-CoV-2. Essa possibilidade enfatiza a importância da obtenção de pelo menos dois resultados negativos no teste de reação em cadeia da polimerase via transcriptase reversa para SARS-CoV-2. O emprego de ensaios sorológicos não foi de grande utilidade no caso relatado, pois a paciente apresentava baixos níveis de anticorpos no final do período de acompanhamento. Baixas cargas virais podem não ser detectadas pelos métodos moleculares vigentes, levando a conclusões equivocadas quanto à eliminação do vírus.

Dado o colapso hospitalar catastrófico, novos estudos de coorte, com caracterização clínica e laboratorial dos pacientes com COVID-19, e incluindo resultados repetidos em testes para detecção de RNA de SARS-CoV-2, são necessários para melhor compreensão e maior eficácia de resposta à pandemia da COVID-19. Quanto melhor for a caraterização do perfil dos pacientes, mais eficazes serão o tratamento e a vigilância epidemiológica da COVID-19 fora do sistema de saúde.
